# Exercise-induced adaptations in the kynurenine pathway: implications for health and disease management

**DOI:** 10.3389/fspor.2025.1535152

**Published:** 2025-03-06

**Authors:** Marcus Vinicius dos Santos Rangel, Karynne Grutter Lopes, Xuebin Qin, Juliana Pereira Borges

**Affiliations:** ^1^ Laboratory of Physical Activity and Health Promotion, Institute of Physical Education and Sports, University of Rio de Janeiro State, Rio de Janeiro, RJ, Brazil; ^2^ Postgraduate Program in Clinical and Experimental Physiopathology, Faculty of Medical Sciences, State University of Rio de Janeiro, Rio de Janeiro, RJ, Brazil; ^3^ Department of Microbiology and Immunology, Tulane National Primate Research Center and Tulane University School of Medicine, Covington, LA, United States

**Keywords:** disease management, tryptophan, chronic disease, kynurenic acid, metabolism, exercise, kynurenine, health promotion

## Abstract

**Background:**

Tryptophan (TRP) metabolism through the kynurenine (KYN) pathway is influenced by inflammatory mediators, generating metabolites that regulate immune and inflammatory responses. Exercise has been proposed as a modulator of this pathway, but its role in health benefits and chronic disease management remains unclear.

**Objective:**

This systematic review examines exercise-induced adaptations in the KYN pathway and their potential implications for health and disease management. Additionally, we identify key methodological considerations for future research.

**Methods:**

A structured search of PubMed/Medline, Web of Science, and Scopus was conducted up to October 2024 to identify clinical trials investigating the effects of exercise training on the KYN pathway.

**Results:**

Of 2,795 articles initially found, 13 clinical trials involving 592 participants met the inclusion criteria. Most studies reported exercise-induced adaptations in the KYN pathway, particularly in cancer survivors. These adaptations appeared to be influenced by exercise intensity and duration. However, several methodological limitations were noted, and no trials included patients with metabolic or cardiovascular diseases.

**Conclusions:**

Here, we show that exercise training modulates the KYN pathway in both healthy and diseased populations, highlighting its potential for disease prevention and management. However, further randomized-controlled trials are needed to clarify its mechanisms and clinical applications, particularly in metabolic and cardiovascular diseases.

**Systematic Review Registration:**

https://www.crd.york.ac.uk/PROSPERO/view/CRD42022351481, PROSPERO (CRD42022351481).

## 1 Introduction

Tryptophan (TRP) is an essential amino acid derived entirely from dietary sources required for protein biosynthesis. Discovered by Hopkins and Cole (1) in 1901 and structurally characterized by Ellinger and Flamand (2) in 1907, TRP has since been shown to participate in several metabolic pathways (3). However, only a small percentage of ingested TRP participates in protein biosynthesis; more than 95% is broken down via the kynurenine (KYN) pathway (4–6), producing various metabolites that have significant roles in regulating immune responses, inflammation, neuronal functions, and gut homeostasis (7–9). These metabolites, collectively referred to as KYN, include kynurenine (KYN), kynurenic acid (KYNA), and quinolinic acid (QUINA) (10), and are involved in the production of nicotinic acid, a precursor for nicotinamide adenine dinucleotide (NAD) (3), which is crucial for cellular energy metabolism (11). Except for hepatocytes, few cells have enzymatic apport to fully degrade TRP to NAD. This makes KYN metabolites important mediators of crosstalk between cells or organs, as they can be exchanged between tissues to exert various biological effects (12, 13).

**Figure 1:**
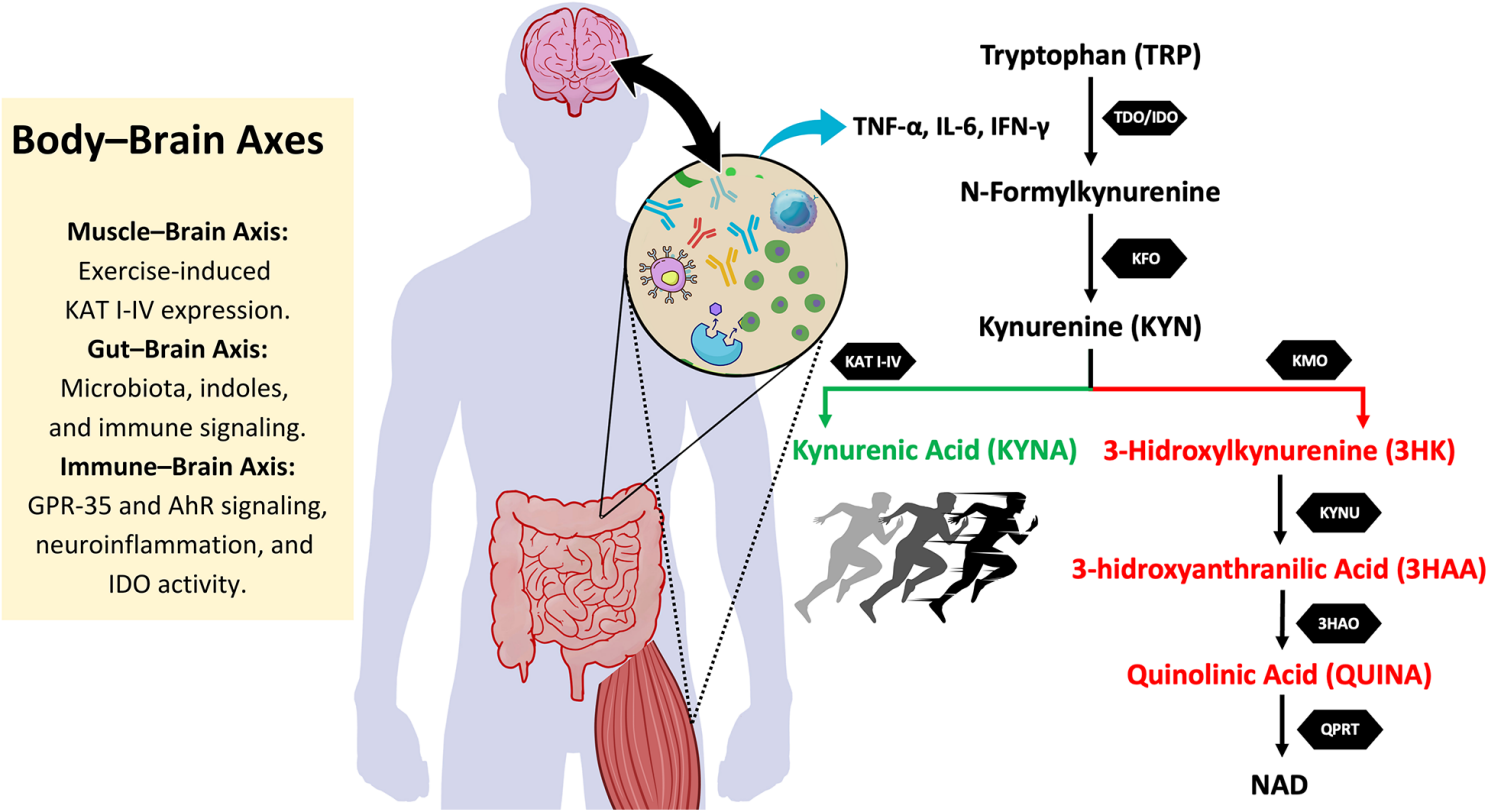
Body-Brain axes and its interaction with the kynurenine pathway. This figure illustrates the kynurenine (KYN) pathway and its interaction with different body-brain axes. Tryptophan (TRP) metabolism is initiated by the enzymes tryptophan 2,3-dioxygenase (TDO) and indoleamine 2,3-dioxygenase (IDO), leading to the formation of N-formylkynurenine, which is further converted into kynurenine (KYN). From this point, KYN follows two main metabolic routes: the neuroprotective pathway (in green), where kynurenine aminotransferases (KAT I-IV) convert KYN into kynurenic acid (KYNA), a metabolite with anti-inflammatory and neuroprotective properties, and the neurotoxic pathway (in red), in which kynurenine 3-monooxygenase (KMO) converts KYN into 3-hydroxykynurenine (3HK). 3HK is further metabolized into 3-hydroxyanthranilic acid (3HAA) and then into quinolinic acid (QUINA), a neurotoxic compound involved in excitotoxicity and neuroinflammation. Exercise promotes KAT expression, favoring KYNA production and shifting the balance towards the neuroprotective pathway. Additionally, the figure highlights key body-brain axes that influence this metabolism. The muscle-brain axis is involved in exercise-induced KAT expression, enhancing KYNA levels. The gut-brain axis regulates kynurenine metabolism through microbiota-derived signals and immune modulation. The immune-brain axis plays a crucial role in shifting TRP metabolism, as pro-inflammatory cytokines such as TNF-α, IL-6, and IFN-*γ* stimulate IDO activity, favoring the production of neurotoxic metabolites. Together, these interactions influence neuroinflammation, neurodegenerative processes, and exercise-induced neuroprotection. KFO, kynurenine formamidase; KYNU, kynureninase; 3HAO, 3-hydroxyanthranilic acid oxygenase; QPRT, quinolinate phosphoribosyltransferase; NAD, nicotinamide adenine dinucleotide; AhR, aryl hydrocarbon receptor; GPR35, G protein-coupled receptor 35.

One of the key regulators of the KYN pathway are two enzymes: indoleamine 2,3-dioxygenase (IDO1) and tryptophan 2,3-dioxygenase (TDO). IDO1 is expressed in a wide range of tissues, including the brain, lungs, heart, kidneys, and intestines, while TDO is primarily active in the liver (14). Both enzymes catalyze the initial step of TRP catabolism, converting TRP into formylkynurenine (14), which is further metabolized to KYNA via kynurenine aminotransferases (KAT) or to 3-hydroxykynurenine (3HK) via kynurenine 3-monoxygenase (KMO), and eventually to QUINA (14, 15). Figure 1 illustrates the KYN pathway.

The activity of IDO1 and TDO increases in response to cytokine signaling, particularly during inflammatory responses (16, 17). Consequently, the KYN pathway is tightly regulated by cytokines, which can either enhance or suppress its activity depending on the body's inflammatory state (18). In chronic conditions, sustained elevation of inflammatory cytokines leads to excessive activation of this pathway (19–21). This overactivation leads to an increase in the production of neurotoxic metabolites, such as 3HK and QUINA (see Figure 1). 3HK and QUINA exert neuronal excitotoxicity due to its agonist activity at N-methyl-D-aspartate receptors (NMDAR) (22). These metabolites contribute to inflammation, immune tolerance, oxidative stress, and neuronal apoptosis (3, 12, 23, 24), and are implicated in the pathogenesis of several diseases, including neurodegenerative disorders (such as Alzheimer's, Parkinson's, and multiple sclerosis) (3, 25, 26) and cancers (24, 27, 28), both of which show elevated KYN levels. Additionally, aging is associated with alterations in the KYN pathway, as increased levels of KYN were observed in older muscle tissues (29). Elevated KYN levels are also correlated with several metabolic disorders, including obesity, dyslipidemia, insulin resistance, and diabetes (30, 31). In contrast, TRP levels are inversely associated with cardiovascular disease incidence (32, 33).

Despite the harmful effects of KYN metabolites like QUINA (34, 35), the KYN pathway also produces neuroprotective agents, such as KYNA (36, 37). KYNA acts by antagonizing NMDAR and α7 nicotinic acetylcholine receptors (α7nAChR) (38, 39), protecting neurons from excitotoxicity and oxidative damage (40). KYNA also exerts anti-inflammatory effects through its interaction with G protein-coupled receptor 35 (GPR35) in adipocytes, which inhibits TNF-α release by macrophages under inflammatory conditions (41–43). Additionally, KYNA mediates anti-inflammatory responses (44, 45) by activating the aryl hydrocarbon receptor (AhR), which promotes the differentiation of T helper 17 (Th17) cells into regulatory T cells (Treg) (46). These mechanisms suggest that KYNA plays a critical role in maintaining the balance between neurotoxicity and neuroprotection within the KYN pathway (47, 48).

The peripheral KYN pathway also influences the central nervous system (39). While KYN, 3HK, and other metabolites can cross the blood-brain barrier, KYNA and QUINA are generally restricted to peripheral tissues (49). This restriction raises the possibility that altering the balance of KYN metabolism in peripheral tissues, for instance by increasing KYNA production, may help reduce the neurotoxic effects of elevated KYN levels in the brain. Given that TRP, KYN, and 3HK can pass through the blood-brain barrier, strategies aimed at rerouting the KYN pathway toward KYNA production could theoretically provide a therapeutic approach to mitigating neurodegenerative diseases and other central nervous system disorders (39, 50).

Lifestyle-based interventions have recently been suggested to modulate TRP metabolism, aiding in the prevention and treatment of diseases with inflammatory mechanisms (39, 44). Exercise training, in particular, has been shown to increase the expression of KAT, redirecting the KYN pathway towards its protective branch in skeletal muscle in humans (51, 52) and mice (41, 51, 53). Evidence from pre-clinical models shows that this re-routing enhances lipid metabolism, and thermogenesis, and reduces weight gain, inflammation, insulin resistance, and glucose intolerance (41, 54), although energy metabolism was largely unaffected in KMO knockout mice (55). Additionally, clinical evidence supports the beneficial role of physical exercise on the KYN pathway in cancer (56, 57) and central nervous system disorders, such as major psychological disorders (39, 58–60). Conversely, studies in healthy individuals (61) and older adults at risk of dementia have failed to identify changes in KYN pathway and benefits after exercise training (60). Collectively, these findings suggest that the benefits of exercise may be more pronounced in certain populations or disease states. There is also growing interest in the role of exercise-induced adaptations of the KYN pathway in chronic diseases associated with inflammation, such as metabolic disorders (62, 63).

Given the potential exercise-induced adaptations in the KYN pathway and their implications for chronic diseases, further research is needed to elucidate the effects of exercise training and its mechanisms on KYN pathway, and to determine whether these effects translate into meaningful clinical benefits for individuals with different health conditions. This review systematically examines clinical trials investigating the adaptations to exercise training on the KYN pathway and its impact on health and disease. We first explore how physical exercise influences this pathway, discussing the molecular adaptations that may contribute to its protective effects in healthy populations. Next, we provide an overview of the findings of the exercise-induced adaptations on the KYN pathway in various chronic conditions. We then summarize findings, identifying key methodological considerations that may explain discrepancies in literature. Finally, we outline current knowledge gaps and propose future research directions to enhance our understanding of how exercise modulates TRP metabolism and whether these adaptations translate into meaningful clinical benefits.

## 2 Methods

This systematic review was conducted in accordance with the Preferred Reporting Items for Systematic Reviews and Meta-Analysis Protocols (PRISMA) guidelines (64). The study was registered in the International Prospective Register of Systematic Reviews (PROSPERO) under the number CRD42022351481, and the protocol was strictly followed through all stages of this review. Studies were selected according to the criteria mentioned in the below sections.

### 2.1 Search strategy

Searches were conducted from inception until August 5, 2022, and updated on October 25, 2024, in MEDLINE (*via* PubMed), Web of Science, and Scopus databases. No date restrictions were applied, and filters were set for human studies and English language articles. A search strategy using Boolean operators “AND” and “OR” and terms related to “exercise training” and “kynurenine pathway” was applied to identify relevant trials (see Supplementary Appendix 1).

### 2.2 Study selection

After removing duplicates, two independent investigators (MR and JB) screened studies in two stages: (1) title and abstract review, and (2) full-text evaluation. Studies failing to meet inclusion criteria at any stage were excluded. Reference lists of selected studies were manually reviewed for additional eligible studies. Discrepancies were resolved through discussion between investigators, and if consensus could not be reached, a third reviewer (KGL) was consulted. Agreement on inclusion was validated in a random sample of 50 abstracts, yielding a Cohen's kappa coefficient of 0.84–0.99 (*p* < 0.05).

### 2.3 Eligibility criteria

Only original trials investigating the effects of exercise training on KYN pathway metabolites were included. Studies were considered if they met the PICOS criteria, as shown in Table 1. No minimum exercise prescription was required. However, authors should have at least reported three of the variables of exercise training prescription, according to the FIIT principle. This principle, which stands for Frequency, Intensity, Time, and Type of exercise, is a fundamental framework used in exercise prescription and research to describe and standardize exercise interventions (65). Each component helps ensure that exercise regimens are clearly defined, reproducible, and comparable across studies.

**Table 1:** Inclusion and exclusion criteria based on PICOS strategy (population, intervention, comparison, outcome and study).

Category	Inclusion criteria	Exclusion criteria
**P**opulation	Adults (≥ 18 years)	Pre-clinical models
**I**nterventions	Supervised exercise training reporting at least three of the FITT principles:	Trials involving dietary or supplementation interventions affecting the KYN pathway
	**F**requency: how often **I**ntensity: how hard	
	**T**ime: duration	
	**T**ype: mode of exercise	
**C**omparison	Pre-post intervention, trained *vs*. untrained	
**O**utcome	Evaluation of at least two KYN pathway metabolites before and after intervention	
**S**tudy	Controlled or non-controlled trials	Acute interventions studies, case reports, epidemiological studies, reviews, and editorials

KYN, kynurenine.

### 2.4 Quality and risk of bias assessment

All included studies were assessed for methodological quality using the Tool for Assessment of Study Quality and Reporting in Exercise (TESTEX scale) (66, 67). TESTEX is a widely used 15-point scale (5 points for study quality and 10 for reporting), specifically designed for exercise studies, addressing criteria not considered in other quality assessment tools. It was chosen due to its validation in evaluating exercise intervention trials and its ability to capture the nuances of exercise prescription fidelity (66).

Additionally, studies were assessed for risk of bias using the Cochrane Collaboration's (RoB, Risk of Bias 2) tool (67, 68). This tool evaluates five domains of bias: Randomization process, Deviations from intended interventions, Missing outcome data, Measurement of the outcome, Selection of the reported result, and Overall bias. Assessments were independently conducted by two authors (MR and JB), and mean scores were assigned for each evaluation method.

## 3 Results

**Figure 2:**
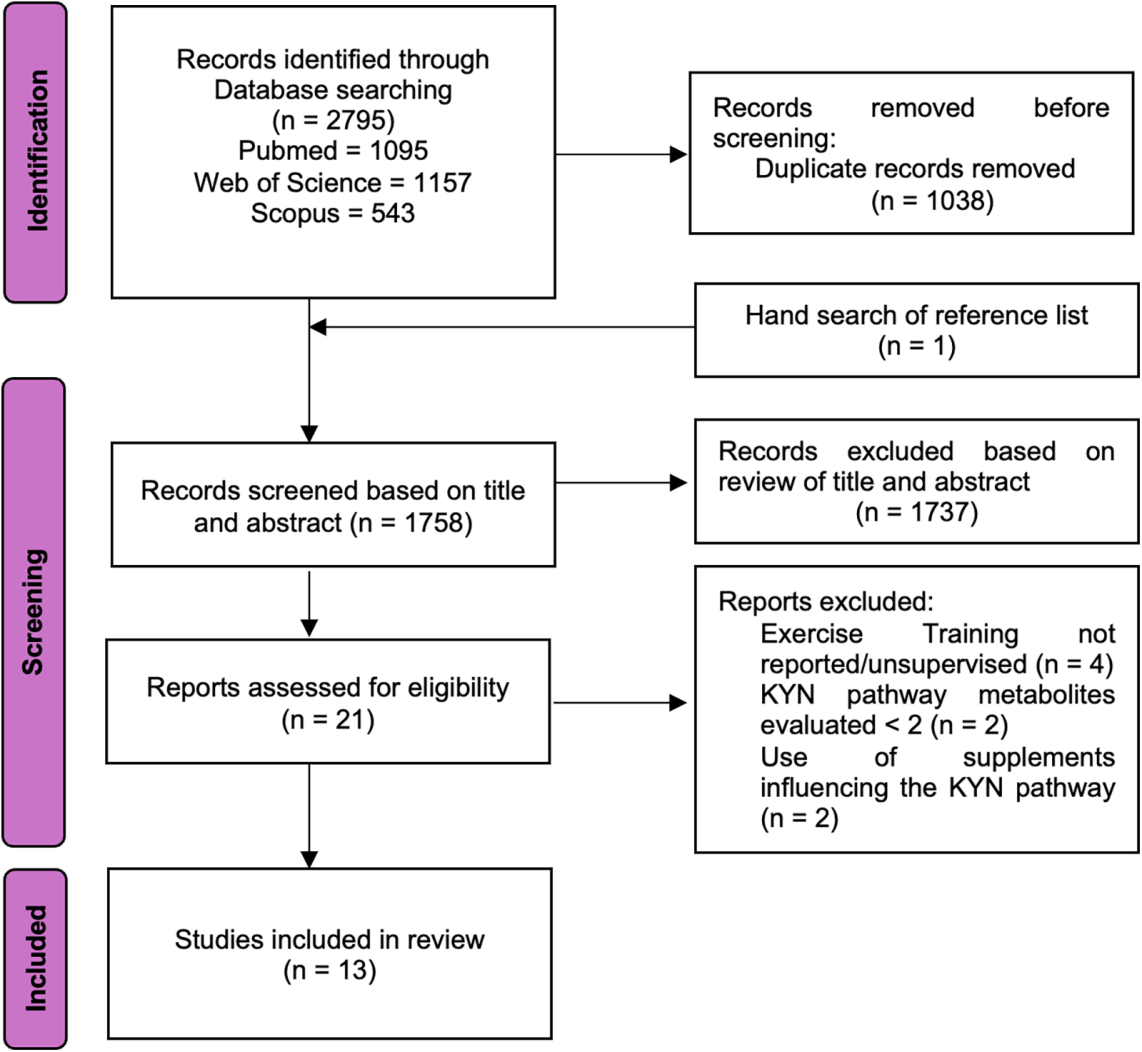
Flowchart summarizing the search and selection of articles.

**Figure 3:**
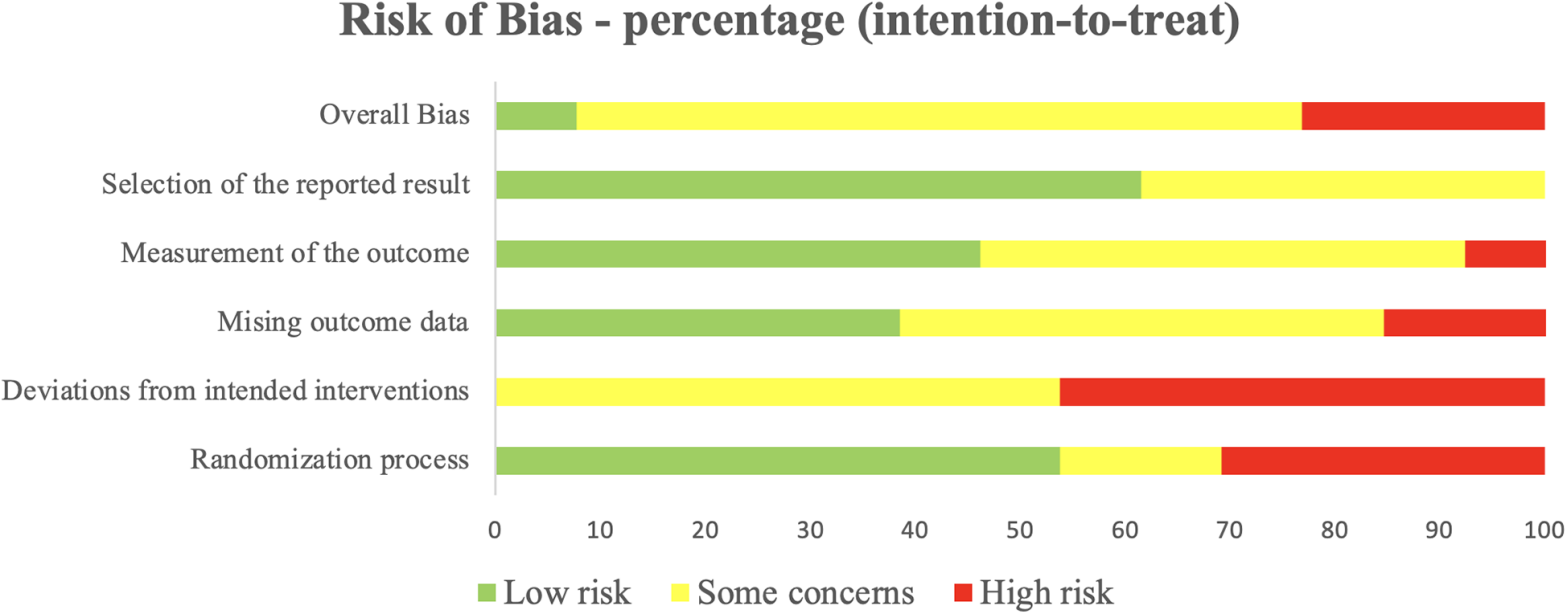
Percentage of studies examining the efficacy of exercise training in modulating the kynurenine pathway with low, some concerns, and high risk of bias for each feature of the cochrane risk of bias tool.

Figure 2 displays the PRISMA flowchart summarizing article selection, while Table 2 presents the methodological quality scores based on TESTEX. Of the 2,796 articles initially found in databases and reference list, 1,038 duplicates were removed, and 1,737 were excluded after title and abstract screening, leaving 21 articles for full evaluation. Of those, 13 articles met the inclusion criteria. Overall, study quality ranged from poor to moderate, with TESTEX scores between 3 and 11 (median score 7). Two trials were rated as high quality (73% of items satisfied), 4 as moderate (50%–72% of items satisfied), and 7 as very low quality (satisfying less than 50% of the items). The risk of bias assessment for each study is presented in Supplementary Appendix 2, with a summary provided in Figure 3. The assessment revealed that most of the included studies (9 out of 13) had some concerns regarding bias. Three studies were classified as having a high risk of bias, while only one was deemed to have a low risk after evaluation.

**Table 2:** TESTEX assessment of the quality and reporting of studies about adaptations to exercise training on the kynurenine pathway in health and disease.

Study	Study quality criterion					Study reporting criterion							∑ (MAX 15)
	1	2	3	4	5	6	7	8	9	10	11	12	
Sánchez et al. (69)	0	0	0	0	0	0	0	2	1	0	0	0	3
Wyckelsma (70)	0	0	1	0	0	3	1	1	1	0	1	1	9
Kamandulis et al. (61)	1	0	0	0	0	0	0	0	1	1	0	0	3
Robbins et al. (56)	1	1	1	0	0	3	0	2	1	0	1	1	11
Pal et al. (57)	1	0	0	1	0	0	0	2	1	0	0	1	6
Pal et al. (71)	1	1	1	1	0	0	0	2	1	0	0	0	7
Zimmer et al. (72)	1	1	1	1	1	1	0	2	1	0	1	1	11
Herrstedt et al. (73)	1	0	1	1	0	2	0	2	1	0	1	0	9
Joisten et al. (74)	1	0	1	0	1	0	0	2	1	0	0	1	7
Bansi et al. (75)	0	0	0	1	0	0	0	2	1	0	0	0	4
Javelle (58)	1	1	1	0	0	1	1	1	1	0	0	1	8
Kuster (60)	1	0	1	1	1	2	0	1	1	0	0	0	8
Saran (76)	1	0	0	0	0	0	0	0	1	0	0	1	3

∑, Sum of all criterions.

Table 3 summarizes the characteristics of the included studies, such as sample, interventions, and outcomes. Of the 13 studies, 5 were randomized controlled trials, and 8 were non-randomized experiments. Eleven studies (84.6%) were conducted in Europe (57, 58, 60, 61, 70–76), with the remaining 2 in the Americas (56, 69). Regarding study populations, only three studies (23%) involved healthy volunteers (61, 69, 70), while 10 (77%) focused on patients with chronic conditions, including cancer (56, 57, 71–73), multiple sclerosis (74, 75), emotionally impulsivity (58), dementia risk (60), and chronic low back pain (76).

**Table 3:** Summary of studies included in the systematic review.

Study	Sample	Exercise training (FITT)	Outcomes			Conclusions
			**Within Analysis**	**Within Analysis**	**Between Analysis**	
Sánchez et al. (69)	Healthy military men:	Frequency: 2 days/wk (dry-land) + 3∼4 days/wk (tactical training)	Divers:	Swimmers:	Divers *vs* Swimmers	Swimming showed a shift in circulating TRP metabolites in relation to diving and sedentarism.
	ExT divers (*n* = 20);		TRP↔	TRP↓	TRP ↓	
			KYN↔	KYN↔	KYN ↑	
	ExT rescue swimmers (*n* = 14);	Intensity: 60%–80% HRmax	KYNA↔	KYNA↔	KYNA ↓	
		Time: 20 min (dry-land) + 90 min (tactical training), 6 months	3HK↑	3HK↔	3HK ↓	
	Untrained controls (*n* = 12)	Type: dry-land (aerobic + strength exercises) + tactical training (diver's or rescue swimmers')	KYN/TRP↔	KYN/TRP↔		
Wyckelsma et al. (70)	Active male older adults:	Frequency: 3 days/wk	Placebo ±ExT:	Antioxidants ±ExT:	Antioxidant *vs*Placebo:	KP metabolism was shifted towards neuroprotection after three weeks of ExT in elderly men, and this shift was blocked by antioxidant treatment.
	Placebo + ExT (*n* = 9)	Intensity: 4–6 reps of 30 s all-out cycling bouts with 4 min of rest	TRP↔	TRP↔	TRP↔	
	Antioxidant vitamin C and E + ExT (*n* = 11)		KYN↔	KYN↔	KYN↔	
		Time: ∼30 min, 3 wks	KYNA↔	KYNA↔	KYNA↔	
		Type: sprint interval training	3HK↔	3HK↔	3HK↔	
			QUINA↓	QUINA↔	QUINA↔	
			PA↔	PA↔	PA↔	
			KYN/TRP↔	KYN/TRP↔	KYN/TRP↔	
			KYNA/QUINA↑	KYNA/QUINA↔	KYNA/QUINA↔	
			KAT I↔	KAT I↔	KAT I↔	
			KAT III↑	KAT III↔	KAT III↔	
			KAT IV↔	KAT IV↔	KAT IV↔	
			TDO2↔	TDO2↔	TDO2↔	
Kamandulis et al. (61)	Healthy Adults (*n* = 20)	Frequency: 3 or 6 days/wk.	KYN↔	NA	NA	ExT over 3 weeks did not induce changes in the concentration of metabolites in the KYN pathway.
		Intensity: Resistance: NR; HIIT: 0.75 Nm/kg.	KYNA↔			
		Time: Resistance: 3 sets of NR repetitions for 3 exercises; HIIT: 4 to 6 30 s all-out cycling sets at 0.75 Nm/kg body weight on a bicycle ergometer with 4 min of rest, 3 wks.	3HK↔			
			QUINA↔			
		Type: Resistance or Resistance + HIIT.				
Robbins et al. (56)	Breast cancer survivors:	Frequency: 3 days/wk	ExT:	Untrained Controls:	ExT *vs*. Untrained:	Therapeutic effects of ExT for breast cancer survivors are mediated through the activation of PGC-1α, leading to changes in KYN metabolism
	ExT (*n* = 22);	Intensity: 2 sets x 15 RM + 1 set to exhaustion (7 major muscle groups)	KYN↓	KYN↔	KYN↓	
			KYNA↔	KYNA↔	KYNA↔	
			KYN/KYNA↓	KYN/KYNA↔	KYN/KYNA↓	
	Untrained controls (*n* = 10)	Time: NR, 12 wks	PGC-1α↑	PGC-1α↔	PGC-1α↑	
		Type: strength training				
Pal et al. (57))	Breast and prostate cancer survivors:	SET	SET:	PET:	SET *vs*. PET:	Aerobic training regulates AhR/IDO axis
		Frequency: 2 days/wk	AhR↔	AhR↔	AhR↔	
	Standard endurance training (SET: *n* = 9);	Intensity: 97% AT	IDO↑	IDO↓	IDO↑	
		Time: 30 min, 12 wks				
		Type: cycling				
	Polarized endurance training (PET: *n* = 12)	PET				
		Frequency: 1 day/wk MICT + 1 day/wk HIIT				
		Intensity: at first lactate threshold (MICT) or 4 × 4 min at 85%–95% HRmax with 3 min of rest (HIIT)				
		Time: individually prescribed (MICT) or ∼25 min (HIIT), 12 wks				
		Type: cycling				
Pal et al. (71)	Pancreatic cancer survivors:	Frequency: 2 days/wk	Supervised ExT:	Home-based ExT:	Supervised *vs*. Home-based	Supervised strength training downregulates the KTR (IDO/TDO) levels and may reduce possible disease progression in pancreatic cancer patients under chemotherapy
	Supervised ExT (*n* = 7);	Intensity: 60%–80% 1RM (supervised) or Borg 14–16 (home-based)	KYN↔	KYN↑		
			TRP↔	TRP↔	KYN↓	
	Home-based ExT (*n* = 14)		KYN/TRP↔	KYN/TRP↑	TRP↔	
		Time: NR, 6 months	Controls:		KYN/TRP↓	
	Control group (*n* = 11)	Type: strength training	KYN↔			
			TRP↔			
			KYN/TRP↔			
Zimmer et al. (72)	Breast cancer survivors:	Frequency: 2 days/wk	ExT:	Healthy women:	ExT *vs*. Healthy women:	Resistance training reduces KYN levels in breast cancer survivors under radiotherapy
	ExT (*n* = 52);	Intensity: 3 × 12 RM (60%–80% 1RM)	TRP↔	TRP↔		
	Untrained (*n* = 44);	Time: 60 min, 12 wks	KYN↓	KYN↔	TRP↔	
			KYNA↔	KYNA↔	KYN↔	
	Healthy women (*n* = 24)	Type: strength training	QUINA↔	QUINA↓	KYNA↔	
			KYN/TRP↔	KYN/TRP↔	QUINA↑	
			KYNA/KYN↓ QUINA/KYNA↑	KYNA/KYN↔ QUINA/KYNA↓	KYN/TRP↔	
					KYNA/KYN↔ QUINA/KYNA↑	
			Untrained:			
			TRP↔		ExT *vs*. Untrained:	
			KYN↑		TRP↔	
			KYNA↔		KYN↓	
			QUINA↔		KYNA↔	
			KYN/TRP↑		QUINA↔	
			KYNA/KYN↓ QUINA/KYNA↔		KYN/TRP↓	
					KYNA/KYN↔ QUINA/KYNA↔	
Herrstedt et al. (73)	Gastro-esophageal junction cancer survivors:	Frequency: 2 days/wk	ExT:	Untrained:	ExT *vs*. Untrained:	Supervised ExT attenuated inflammatory and neuroexcitatory metabolites
		Intensity: NR	TRP↓	TRP↓	KMO↓	
	ExT (*n* = 18);	Time: 30–45 min, 12 wks	KYN↔	KYN↔		
		Type: cycling, strength training	KYNA↔	KYNA↔		
	Untrained (*n* = 5)		QUINA↔	QUINA↑		
			3HK↔	3HK↑		
			XA↔	XA↔		
			AA↑	AA↑		
Joisten et al. (74)	Multiple Sclerosis:	Frequency: 3 days/wk	NR	NR	MICT *vs*. HIIT:	The KYN/TRP upregulation following 3 weeks of HIIT suggests disease-counterregulatory properties of exercise on immune homeostasis, which remains to be investigated.
	MICT (*n* = 34);	Intensity: 65% Hrmax (MICT) or 5 × 1.5 min at 95–100% Hrmax with 2 min recovery			IL-6↔	
	HIIT (*n* = 35)				TRP↔	
		Time: 30 min (MICT) or ∼22 min (HIIT), 3 wks			KYN↔	
					QUINA↔	
		Type: cycling			KYNA↔	
					QUINA/KYN↔	
					KYNA/KYN↔	
					QUINA/KYNA↔	
					KYN/TRP↓	
Bansi et al. (75)	Multiple Sclerosis:	Frequency: 3 days/wk	SPMS:	RRMS:	SPMS *vs*. RRMS:	MS subtypes have different KP responses to ExT
	Secondary progressive HIIT (SPMS HIIT; *n* = 11);	Intensity: 5 × 3 min at 85%–90% HRmax with 1.5 min recovery	TRP↑	TRP↓	TRP↔	
			KYN↔	KYN↔	KYN↔	
			KYN/TRP↓	KYN/TRP↑	KYN/TRP↓	
	Secondary progressive MICT (SPMS MICT; *n* = 13);	Time: 20 min, 3 wks			No differences found between the training modalities (HIIT vs. MICT)	
		Type: cycling				
	Relapsing remitting HIIT (RRMS HIIT; *n* = 16);					
	Relapsing remitting MICT (RRMS MICT; *n* = 17)					
Javelle et al. (58)	Emotionally impulsive humans:	Frequency: 3 days/wk	HIIT:	Stretching:	HIIT *vs*. Stretching:	HIIT reduced the IL-6 levels and the neurotoxic branch of the KP
		Intensity: 4 × 4 min at 85%–95% HRmax with 3 min recovery	KYN/TRP↔	KYN/TRP↔	QUINA/KYN↓	
	HIIT (*n* = 28);		KYNA/KYN↔	KYNA/KYN↔	KYNA/QUINA↑	
			QUINA/KYN↓	QUINA/KYN↔	IL-6↓	
	Control stretching (*n* = 25)	Time: 30 min, 8 wks	KYNA/QUINA↑	KYNA/QUINA↔		
		Type: aerobic exercise or stretching	IL-6↓	IL-6↔		
Küster et al. (60)	Older adults at risk of dementia:	Frequency: 5 days/wk (2 at center + 3 at home)	ExT:	Cognitive Training:	Cognitive Training *vs*. ExT:	Associations of irisin and metabolites of the KP with BDNF and cognition on the one hand, and with psychosocial stress as well as cognitive or physical training on the other hand, indicate that these biological measures may constitute candidate mediators of lifestyle influences on cognition and dementia in old age
			KYN↔	KYN↔		
	ExT (*n* = 21);	Intensity: NR	KYNA↔	KYNA↓	KYN↔	
			3HK↔	3HK↓	KYNA↔	
	Cognitive training (*n* = 18);	Time: 60 min (center) or 20 min (home), 10 wks	QUINA↔	QUINA↔	3HK↓ (Cognitive group)	
			Untrained controls:			
	Untrained controls (*n* = 25)				QUINA↔	
		Type: Aerobic, coordination, balance, stretching, strength training	KYN↔			
			KYNA↔			
			3HK↔			
			QUINA↔			
Saran et al. (76)	chronic low back pain patients (*n* = 35)	Frequency: 5 days/wk	After 2 wks of ExT:	After 4 wks of Ext:	NA	A two-week cycle of physical exercise decreased the KYN and increased KYNA content in sweat. Physical exercises result in a long-term increase in the KAT activity responsible for the formation of KYNA from KYN.
		Intensity: 85% HRmax		TRP↔		
		Time: 16–30 min (progressively), 4 wks	TRP↔	KYN↔		
		Type: cycling, elliptical cross-training	KYN↓	KYNA↔		
			KYNA↑	KAT↑		
			KAT↑	IDO/TDO↓		
			IDO/TDO↓			

RM, repetition maximum; ExT, exercised trained; reps, repetitions; HRmax, maximal heart rate; AT, anaerobic threshold; MICT, moderate intensity continuous training; HIIT, high intensity interval training; NA, not apply; NR, not reported; KP, kynurenine pathway; TRP, tryptophan; KYN, kynurenine; KYNA, kynurenic acid; 3HK, 3-Hydroxykynurenine; QUINA, quinolinic acid; KAT, kynurenine aminotransferase; IDO, indoleamine 2,3-dioxygenase; TDO, tryptophan-2,3-dioxygenase; PGC-1α, peroxisome proliferator-activated receptor-gamma coactivator-1 alpha; AhR, aryl hydrocarbon receptor; XA, xanthurenic acid; AA, anthranilic acid; KMO, kynurenine-3-monooxygenase; IL-6, interleukin-6.

Among the 13 included studies, 11 reported evidences of exercise-induced adaptations in the KYN pathway (56–58, 69–76). The two studies (60, 61) that failed to observe such adaptations were conducted in healthy individuals (61) (representing 33% of all studies in healthy populations), and in older adults at risk of dementia (60). Exercise-induced adaptations included changes in muscle KAT content (70), Peroxisome proliferator-activated receptor-gamma coactivator **(**PGC)-1α (56), KYN or TRP (56, 57, 69, 72, 74–76), KYNA (58, 69, 76) and IDO-1 and 2 levels (71, 76).

## 4 Discussion

We investigated the effects of exercise training on the KYN pathway and its implications in health and chronic conditions. Our findings suggest that exercise-induced adaptations in the KYN pathway differ across populations, with more pronounced effects observed in individuals with chronic diseases. These results contribute to the growing evidence that physical exercise modulates TRP metabolism, promoting neuroprotective and anti-inflammatory effects. Given the increasing interest in the role of the KYN pathway in various pathophysiological conditions, our study provides relevant insights into its responsiveness to exercise interventions.

Limited studies have examined the effect of supervised exercise training on KYN pathway metabolites in healthy individuals. While some research suggests beneficial adaptations (69, 70), findings remain inconsistent (61). In young adults, endurance-based swimming training showed greater reductions in circulating KYN and increases in KYNA compared to tactical immersion training, likely due to differences in oxidative stress and metabolic demands (69). Studies on older adults demonstrated that vigorous sprint interval training significantly reduced plasma QUINA levels and increased the KYNA/QUINA ratio and KAT content (70). However, when exercise was combined with dietary antioxidants, these effects were blunted, suggesting that a pro-oxidant environment may be necessary to drive beneficial shifts in KYN metabolism (70). Additionally, Boßlau, Wasserfurth (77) reported that 12 weeks of unsupervised combined training could redirect the KYN pathway toward KYNA. This shift appears to be associated with mitigating immune senescence in older adults, as evidenced by attenuated CD8+ T-cell differentiation.

Collectively, these findings indicate that exercise intensity plays a critical role in driving KYN pathway adaptations, likely through its influence on oxidative stress and inflammatory signaling. Alongside this finding, another study (78) demonstrated that 4 weeks of unsupervised moderate-intensity home-based exercises failed to improve TRP or KYN pathway in healthy young adults. The authors speculated that a more vigorous exercise regimen would likely have promoted changes in the KYN pathway. However, Kamandulis, Lukonaitiene (61) reported unchanged KYN metabolites after three weeks of combined resistance and high intensity interval training (HIIT), despite improvements in mood profile. Thus, results remain inconsistent, underscoring the need for further research exploring different exercise modalities, including resistance training and HIIT, to determine their impact on KYN metabolism in healthy populations.

The KYN pathway plays a crucial role in immune and neurological regulation (79), and its dysregulation is associated with numerous diseases, including neurodegenerative disorders (80), cancer (81), and metabolic syndrome (82). Chronic inflammation and oxidative stress contribute to pathway overactivation (83), leading to the accumulation of neurotoxic metabolites such as QUINA and 3HK (55). Exercise training appears to counteract these effects by promoting a shift toward KYNA production (54), which exerts neuroprotective and anti-inflammatory properties. Our analysis showed that exercise-induced increases in KYNA and reductions in the KYN/TRP ratio were more consistent in clinical populations, suggesting that individuals with systemic inflammation may experience greater therapeutic benefits from exercise interventions.

Most research investigating exercise-induced KYN pathway adaptations has focused on cancer survivors, particularly in those with pancreatic (57), gastro-esophageal junction (73), prostate (71), and breast cancers (56, 72) (71). Elevated KYN levels are linked to poor prognosis in cancer patients (84). KYN and its metabolites suppress T-cell function, promote regulatory T-cell differentiation, and impair natural killer cell activity (39, 57, 85). Additionally, NAD + synthesis via the KYN pathway fuels oncogenic processes, as cancer cells rely heavily on NAD + to meet increased ATP demands (24).

Exercise has been shown to reduce cancer risk and progression (86), partly by improving the anti-inflammatory profile and reducing systemic inflammation (87). Resistance training and HIIT have demonstrated benefits in modulating KYN metabolism, likely through exercise-induced activation of PGC-1α, which increases skeletal muscle KAT content (54) and shifts the KYN pathway toward KYNA production (21). This helps to mitigate inflammation by activating GPR35 (41) and the KYNA-AhR axis (12). Additionally, increased KAT levels reroute the KYN pathway, preventing the overproduction of immunosuppressive intermediate metabolites, such as anthranilic acid (AA), 3-hydroxylanthranilic acid (3HAA) and QUINA, which promote immune evasion and cancer cell migration (24, 88). Studies in breast cancer survivors reported reduced KYN levels following 12 weeks of resistance training, with untrained controls exhibiting a shift toward neurotoxic KYN metabolites (34). Similar benefits were observed in pancreatic cancer survivors undergoing chemotherapy, where strength training prevented increases in KYN levels and the KYN/TRP ratio (23). In gastro-esophageal junction cancer survivors, concurrent training attenuated inflammatory and neurotoxic metabolites while reducing depression and anxiety symptoms (35). Interestingly, Robbins, Kelleher (56) reported increased PGC-1α activation following exercise training, suggesting that changes in KYN levels were driven by exercise-induced PGC-1α activation, as supported by animal studies (54).

Regarding the intervention types, Pal, Schneider (71) found HIIT-based training more effective than moderate-intensity continuous training (MICT) in modulating KYN pathway metabolism. Polarized endurance training involving HIIT sessions reduced IDO levels, whereas standard training increased them. Although no changes in AhR levels were observed, the authors suggested that polarized training might downregulate the AhR/IDO axis, affecting natural killers (NK) cells. This is relevant since inflammation-induced increases in IDO elevate KYN, acting as potent AhR agonists in the cancer microenvironment, promoting IDO expression in a feedback loop that suppresses innate immune responses by reducing NK cell function (84). Exercise-induced reductions in IDO, KYN, and AhR expression may therefore enhance immune responses in cancer patients (39, 89).

Neurodegenerative disorders and psychiatric conditions, including depression and schizophrenia, are also linked to KYN pathway dysregulation (90). Javelle, Bloch (58) demonstrated that HIIT reduced inflammation and KYN metabolism in emotionally impulsive individuals, improving impulsivity scores. Exercise also reduced IL-6 levels, possibly via KYNA's anti-inflammatory actions through GPR35 activation (39, 91, 92). In contrast, Küster et al. (60) found no exercise-induced adaptations in KYN pathway metabolism among older adults at risk of dementia. In this study, exercise intensity was not controlled, and only two exercise sessions per week were conducted, which may have limited the potential benefits of the exercise training.

Recently, Kupjetz, Patt (93) conducted a randomized controlled trial comparing the effects of endurance training on KYN pathway modulation in individuals with multiple sclerosis. Their findings indicate that both HIIT and MICT similarly reduced most KYN metabolites over time, with baseline systemic inflammation influencing exercise-induced changes. Likewise, Joisten, Rademacher (74) found no significant differences between HIIT *vs.* MICT for most metabolites, except for an increase in the KYN/TRP ratio. Bansi, Koliamitra (75) also compared these exercise modalities and reported no overall differences, though responses varied by multiple sclerosis subtype. Notably, patients with relapsing-remitting multiple sclerosis, a milder form of the disease, showed an increase in the KYN/TRP ratio compared to those with secondary progressive multiple sclerosis, contradicting the authors' hypothesis that exercise would promote a long-term anti-inflammatory effect. However, these studies employed a three-week intervention, a relatively short duration for promoting chronic adaptations. Additionally, neither study included an untrained control group, making it difficult to determine whether exercise intervention prevented a worsening of KYN metabolism (74). These methodological limitations restrict the generalizability of the findings.

Among all studies examining exercise-induced adaptations in the KYN pathway for disease, only one was conducted outside of cancer or central nervous system disorder populations. Saran, Turska (76) demonstrated that two weeks of aerobic training decreased KYN and increased KYNA levels in patients with chronic low back pain, though these differences were not observed at the end of the protocol (4 weeks). However, the absence of an untrained control group and lack of control over menstrual cycle phases (among women who comprised most of the sample) should be considered. Thus, the promising findings should be interpreted with caution.

While exercise training appears to induce beneficial shifts in KYN metabolism across various conditions (94, 95), critical gaps remain in literature. Notably, no studies have investigated exercise training's potential effects on the KYN pathway in metabolic or cardiovascular diseases (62, 96), despite strong evidence linking KYN dysregulation to conditions such as diabetes and atherosclerosis (97–100). Evidence in this regard only comes from preclinical studies showing positive results (101). Additionally, inflammation-driven diseases, such as HIV (102, 103) and long COVID disease (104, 105), warrant further exploration to determine whether exercise interventions could mitigate disease-related disruptions in KYN metabolism.

Several inconsistencies remain regarding the optimal exercise modalities and intensities required to induce meaningful changes in the KYN pathway (93, 106). High-intensity exercise appears more effective than moderate-intensity training, but further research is needed to establish standardized exercise prescriptions. Additionally, individual factors such as age, sex, genetic predisposition, and baseline inflammatory status likely influence exercise-induced TRP metabolism changes, necessitating a more personalized approach to exercise interventions.

One of the key limitations in this field is the methodological variability across studies in healthy and diseased populations. Differences in sample size, exercise prescription, and biomarker assessment methods contribute to inconsistent findings. Future studies should prioritize well-designed randomized controlled trials (RCTs) with standardized exercise protocols and rigorous analytical techniques to establish causal relationships between exercise and KYN pathway modulation. Additionally, incorporating multi-omics approaches, including transcriptomics and proteomics, could help identify novel regulatory mechanisms underlying exercise-induced metabolic adaptations.

From a clinical perspective, our findings underscore the potential for targeted exercise interventions to mitigate inflammation and neurotoxicity by modulating the KYN pathway. Personalized exercise prescriptions based on metabolic profiling could optimize therapeutic outcomes, and incorporating KYN biomarkers into clinical assessments may provide valuable insights into inflammatory and metabolic status (107, 108), guiding clinical decision-making (109). Moreover, structured exercise programs could serve as non-pharmacological strategies for managing chronic diseases characterized by KYN dysregulation.

## 5 Conclusions and future directions

The evidence suggests that exercise training plays a crucial role in modulating KYN pathway metabolism, particularly in individuals with chronic diseases characterized by low-grade inflammation (23). These conditions often drive KYN metabolism toward neurotoxic metabolites (12, 39), whereas exercise training promotes a shift toward the neuroprotective branch. This effect appears more pronounced in cancer patients due to elevated IDO activity, while findings in central nervous system disorders remain inconsistent, possibly due to methodological variations. Additionally, exercise volume and intensity seem to be key moderators of these benefits.

Despite promising results, few studies have explored exercise-induced KYN pathway adaptations in healthy adults. Additionally, most research has yet to establish direct links between KYN pathway changes and clinical outcomes (110). Future research should bridge this gap by integrating mechanistic insights with clinical relevance endpoints, particularly in metabolic, infectious, and cardiovascular diseases. Experimental models, including animal studies, could provide controlled conditions to help clarify dose-response relationship and underlying pathways. Understanding these mechanisms will enhance the therapeutic potential of exercise and refine its application in clinical settings. By addressing these challenges, future research can solidify the role of exercise in mitigating inflammation-driven neurotoxicity and advancing targeted interventions for vulnerable populations.

## Data Availability

The original contributions presented in the study are included in the article/Supplementary Material, further inquiries can be directed to the corresponding author.
