# Supplementary material for: Exercise-induced adaptations in the kynurenine pathway: implications for health and disease management
Source: Front Sports Act Living. 2025 Mar 6;7:1535152. doi: 10.3389/fspor.2025.1535152 (PMC11922725; doi:10.3389/fspor.2025.1535152)
Supplement: Supplementary file 2 [file Table1.docx]

**Supplementary table 2:** Risk of bias assessment.
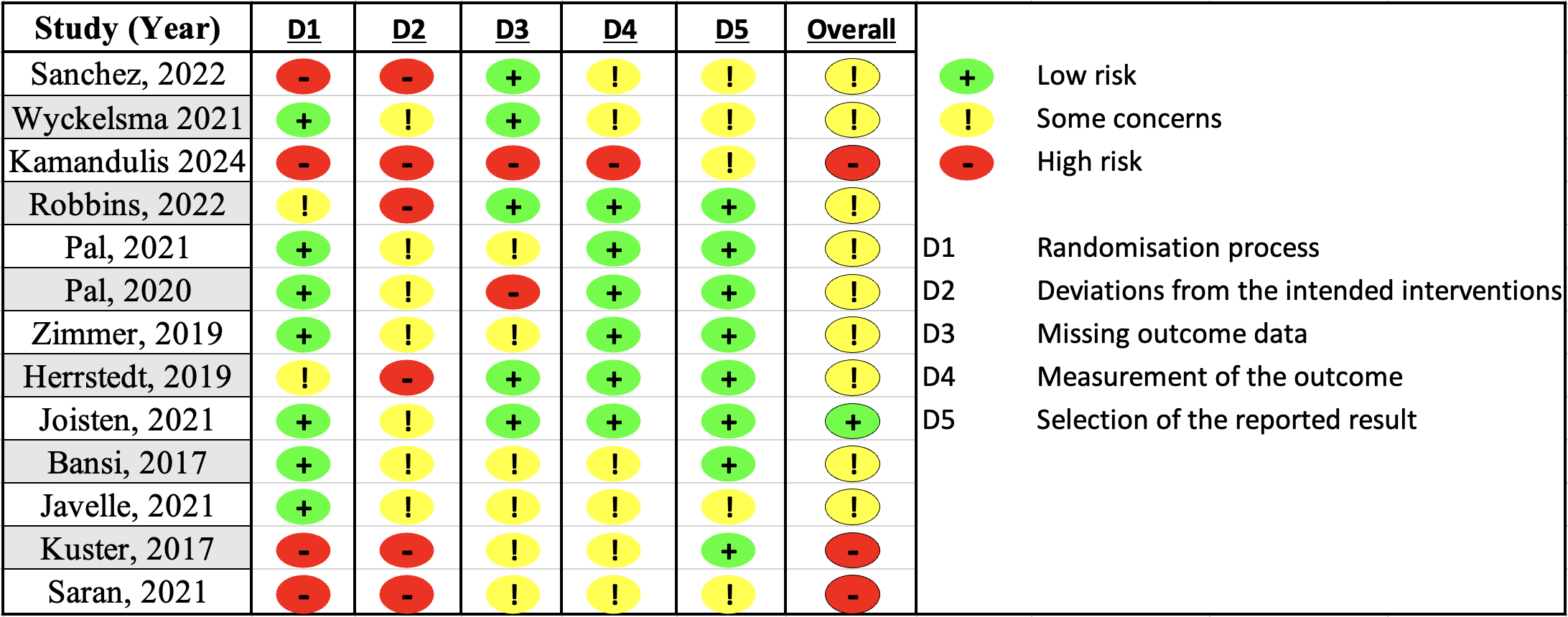


Overall risk-of-bias judgement Criteria:

Low – Low risk of bias The study is judged to be at low risk of bias for all domains for this result.

Some concerns - The study is judged to raise some concerns in at least one domain for this result, but not to be at high risk of bias for any domain.

High- High risk of bias The study is judged to be at high risk of bias in at least one domain for this result. Or The study is judged to have some concerns for multiple domains in a way that substantially lowers confidence in the result.
